# Sitagliptin and risk of heart failure hospitalization in patients with type 2 diabetes on dialysis: A population-based cohort study

**DOI:** 10.1038/srep30499

**Published:** 2016-07-27

**Authors:** Yi-Chih Hung, Che-Chen Lin, Wei-Lun Huang, Man-Ping Chang, Ching-Chu Chen

**Affiliations:** 1 Division of Endocrinology and Metabolism, Department of Medicine, China Medical University Hospital, Taichung 40447, Taiwan; 2 Department of Medicine, China Medical University, Taichung 40402, Taiwan; 3 Management Office for Health Data, China Medical University Hospital, Taichung 40447, Taiwan; 4 Department of Public Health, China Medical University, Taichung 40402, Taiwan; 5 Department of Nursing, School of Health, National Taichung University of Science and Technology, Taichung 40343, Taiwan; 6 School of Chinese Medicine, China Medical University, Taichung 40402, Taiwan

## Abstract

The incidence of heart failure hospitalization (HHF) after taking sitagliptin in type 2 diabetes (T2DM) patients with end stage renal disease (ESRD) on dialysis is unclear. In this population-based cohort study, we identified individuals with T2DM and ESRD on dialysis who were treated with sitagliptin between 2009 and 2011 and randomly selected a control cohort matched by age, sex, duration of T2DM, hypertension medications, use of statin and aspirin, sulfonylureas, glinides, and insulin usage, atherosclerotic heart disease, congestive heart failure and chronic obstructive pulmonary disease at a 1:4 ratio. Multivariable Cox proportional hazards regression analysis was used to evaluate HHF risk. The overall incidence of HHF was higher in the sitagliptin cohort than in the control cohort (1130 *vs*. 754 per 10000 person-years; adjusted hazard ratio (HR): 1.52, 95% CI = 1.21–1.90). There was a significant trend towards increased HHF risk associated with increased sitagliptin dose (*p* for trend < 0.01). Subjects at greater risk of HHF after taking sitagliptin were those without severe hypoglycemia, without ACE inhibitors treatment, with history of heart failure or receiving hemodialysis rather than peritoneal dialysis. In conclusion, use of sitagliptin was associated with an increased risk of HHF in patients with T2DM on dialysis.

Taiwan has the highest prevalence and the third highest incidence of end stage renal disease (ESRD) in the world as of 2011^1^. Among patients with ESRD, type 2 diabetes (T2DM) is the predominant cause and most of them die from cardiovascular (CV) disease^1^. However, no prospective randomized clinical trials have evaluated the effects of glycemic control on CV outcomes in dialysis patients with diabetes, because these patients usually are excluded from such studies^2^. Despite a paucity of evidence showing the efficacy of adequate glycemic control for preventing CV disease in dialysis patients, practice guidelines for diabetes and chronic kidney disease (CKD) suggest that glycemic management may be beneficial in preventing progression of neurologic and retinal outcomes^3^. Adequate control of diabetes in dialysis patients is challenging for many physicians, because measuring the HbA1c level is less precise in the setting of ESRD and there are limited treatment options. Dipeptidyl peptidase-4 (DPP-4) inhibitors have several potential advantages in treating people with CKD as they are associated with a low risk of hypoglycemia and are weight-neutral. In addition, one meta-analysis has shown that DPP-4 inhibitors appear to be especially effective in Asians^4^. However, a large-scale randomized trial among patients with T2DM who are at risk for CV events has shown that saxagliptin has a neutral effect in relation to CV events; nevertheless, its use was found to be associated with a higher incidence of hospitalization for heart failure (HHF)^5^. This increase in heart failure (HF) risk was highest among patients with elevated levels of N-terminal pro B-type natriuretic peptides (NT-proBNP), prior HF, or CKD^6^. The TECOS (Trial Evaluating Cardiovascular Outcomes with Sitagliptin) is a randomized, double-blind trial that enrolled patients with established CV diseases to evaluate the safety of sitagliptin^7^. This trial showed that adding sitagliptin to the regular medication regimen did not appear to increase the risk of HHF^7^. However, the trial excluded patients who had an eGFR<30 mL/min/1.73 m^2^. Therefore, it is improbable that the results of the TECOS trial will provide information about the safety of sitagliptin therapy in patients with ESRD, who are at a high risk for CV disease. Few other studies reported that sitagliptin was well tolerated in T2DM patients with moderate or severe chronic renal insufficiency (eGFR<30 mL/min/1.73 m^2^ including ESRD on dialysis)^8^ or even in those with ESRD receiving dialysis^9^. However, these studies did not designate CV outcomes as the primary endpoint and the small sample size caused limitations in between-group comparisons. In this study, we aim to evaluate the association of sitagliptin treatment with HHF in patients with T2DM and ESRD on dialysis.

## Results

We identified 870 individuals with ESRD who were taking sitagliptin; these individuals formed the sitagliptin cohort. Furthermore, we matched 3480 non-users to the members of the sitagliptin cohort, which formed the control cohort. The demographic characteristics of the sitagliptin cohort and the control cohort are presented in Table 1. Most patients were ≧65 years old, male, receiving hemodialysis, and had diabetes for about 9 years. The mean follow-up duration was about one year. The comorbidity index and comorbidities including ASHD, CHF, CVA/TIA, PVD, COPD, GI bleeding, liver disease, dysrhythmia and cancer of the sitagliptin cohort and the control cohort were similar. About 99% of the patients in both cohorts had hypertension and 77% of the patients had hyperlipidemia. The development of severe hypoglycemia was not significantly different in both cohorts (14.1% *vs*. 13.4%, *p* = 0.63). About 19% of the individuals in both cohorts were being treated with an angiotensin-converting-enzyme inhibitor (ACEI) and 42% of the patients were taking angiotensin receptor blocker (ARB). Approximately 30% of the patients in both cohorts were being treated with statin and 44% of the patients were taking aspirin. About 30% of the subjects in both groups were being treated with sulfonylureas and 56% of individuals had insulin treatment.

As shown in Table 2, the overall incidence of HHF was higher in patients taking sitagliptin than in non-users (1130 *vs*. 754 per 10000 person-years; adjusted hazard ratio (HR): 1.52, 95% CI = 1.21–1.90). Compared to non-users, individuals exposed to low, intermediate, or high-dose sitagliptin did show such an association with 1.35-fold (adjusted HR 1.35, 95% CI = 1.04–1.74), 2.16-fold (adjusted HR 2.16, 95% CI = 1.40–3.35) and 2.57-fold (adjusted HR 2.57, 95% CI = 1.21–5.47) increase in the risk of HHF, respectively. Moreover, there was a significant associated trend towards increased HHF risk with increasing dose of sitagliptin exposure (p for trend < 0.01).

As shown in Table 3, the risk of HHF was higher among sitagliptin users who didn’t have severe hypoglycemia (adjusted HR 1.51, 95% CI = 1.18–1.93) and who were not treated with ACE inhibitors (adjusted HR: 1.61; 95% CI = 1.24–2.08) in comparison with those who had severe hypoglycemia (adjusted HR: 1.50; 95% CI 0.84–2.69) and those who were taking ACE inhibitors (adjusted HR: 1.26; 95% CI 0.78–2.03). Individuals who had a history of HF were associated with an increased risk of HHF after taking sitagliptin (adjusted HR:1.54; 95% CI 1.19–1.98) as compared to patients without prior HF (adjusted HR: 1.37; 95% CI 0.85–2.21). Patients treated with sitagliptin also had a higher associated risk of HHF among those who were receiving hemodialysis (adjusted HR: 1.54; 95% CI 1.21–1.96) rather than those on peritoneal dialysis.

**Figure 1:**
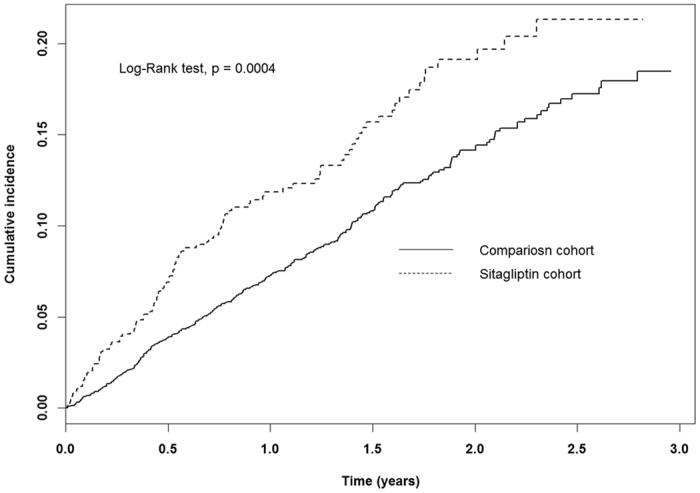
Cumulative incidence of hospitalization for heart failure among patients with type 2 diabetes and ESRD on dialysis, according to sitagliptin use.

Figure 1 shows the cumulative HHF incidence curves for the study cohorts. Among dialysis patients, sitagliptin treatment was significantly associated with a higher risk of HHF as compared to those not using sitagliptin.

## Discussion

To the best of our knowledge, this is the first nationwide, population-based study to evaluate the risk of HHF related to sitagliptin therapy in T2DM patients with ESRD on dialysis. Our study showed that sitagliptin use was associated with an increased risk of HHF in patients with T2DM receiving dialysis, especially in those without severe hypoglycemia, without ACE inhibitors treatment, with prior HF or receiving hemodialysis. In addition, there was a significant trend towards a higher associated risk of HHF as the dose of sitagliptin increased.

Based on FDA recommendations, DPP-4 inhibitors have been tested in large clinical outcome trials: The SAVOR-TIMI 53 (Saxagliptin Assessment of Vascular Outcomes Recorded in Patients with Diabetes Mellitus-Thrombolysis in Myocardial Infarction 53)^5^, the EXAMINE (Cardiovascular Outcomes Study of Alogliptin in Patients with Type 2 Diabetes and Acute Coronary Syndrome)^10^ and the TECOS^7^ trials. The SAVOR trial reports a significantly higher incidence of HHF in patients treated with saxagliptin (3.5% vs. 2.8%; HR 1.27, 95% CI = 1.07–1.51)^5^, which raised the issue of HF in relation to DPP4 inhibitors^11^,^12^. In the EXAMINE trial, although HHF did not achieve statistical significance, there was a numerical increase in HF cases in the alogliptin group (3.9% vs. 3.3%; HR 1.19, 95% CI = 0.90–1.58)^10^. In addition, the VIVIDD (Vildagliptin in Ventricular Dysfunction Diabetes) trial recruited diabetic patients with advanced HF to examine the safety of vildagliptin^13^. This trial suggested that vildagliptin did not adversely reduce left ventricular function; nevertheless, patients with ventricular dysfunction who were treated with vildagliptin did show an increase in left ventricular end-diastolic volume^13^. The mechanisms underlying the potential increased risk of HF upon DPP4 inhibitor use remain unclear. However, despite the data obtained from the SAVOR, EXAMINE, and VIVIDD trials regarding HF cases, the new evidence obtained from the TECOS trial^7^ makes it less very unlikely that the observed increase in HHF seen with saxagliptin is a class effect of DPP4 inhibitors. The TECOS trial showed that the addition of sitagliptin to the conventional pharmacological treatment did not have a significant effect on HHF, as determined after a mean follow-up period of 3 years. A meta-analysis combining the data from the SAVOR, EXAMINE, and TECOS trials showed that the risk of HHF in the DPP4 inhibitor group had not increased (623 cases of HF in the DPP4 inhibitor group vs. 546 in the placebo group; HR, 1.14; 95% CI, 0.97 to 1.34)^14^. However, because these studies excluded patients with ESRD on dialysis, the safety of DPP4 inhibitors were not well characterized in these populations.

Our cohort study has revealed that sitagliptin is associated with an increased risk of HHF among patients with T2DM and ESRD on dialysis. Several potential explanations need to be considered. First, DPP4 inhibitors may cause hypoglycemia, particularly in combination with other hypoglycemic agents. Hypoglycemia stimulates the sympathetic and renin-angiotensin-aldosterone system and chronic stimulation might have adverse results, including progression to sympathetic HF that might require admission to hospital^15^. Rates of hypoglycemia in both the SAVOR and EXAMINE trials were modestly increased in patients taking DPP4 inhibitors. However, despite an increase in the relative risk for hypoglycemia with saxagliptin noted in patients on background sulfonylureas in the SAVOR trial, no increase in the risk of HHF occurred with saxagliptin within this subgroup. Similarly, the differences in hypoglycemia were very minor between the alogliptin and placebo groups in the EXAMINE trial. In the TECOS trial, patients with a history of two or more episodes of severe hypoglycemia (defined as requiring third-party assistance) during the preceding 12 months were excluded and there was no significant difference between the sitagliptin group and the placebo group with respect to hypoglycemia. In our study, the risk of HHF was higher among sitagliptin users who did not have severe hypoglycemia (adjusted HR 1.51, 95% CI = 1.18–1.93) as compared to those who had severe hypoglycemia (adjusted HR: 1.50; 95% CI 0.84–2.69). Taken together, it seems that the presence of hypoglycemia is not the main cause of increased risk of HHF among patients treated with DPP4 inhibitors. Second, Marney *et al*^16^. suggested that sitagliptin interacted with high-dose enalapril to increase rather than decrease blood pressure levels in patients with metabolic syndrome. Furthermore, this interaction was associated with an increase in heart rate and plasma norepinephrine levels that was significant at the highest dose of enalapril. The mechanisms underlying this interaction are unclear but may relate to blockade of the peptides substance P and/or neuropeptide Y with DPP-4 inhibitors, leading to sympathetically mediated vasoconstriction. Similarly, Jackson *et al*^17^. showed that, in a renal perfusion model, enhancement of angiotensin II-mediated constrictor responses due to increasing neuropeptide Y administration could be exacerbated by sitagliptin and blocked if sitagliptin is given along with a neuropeptide Y inhibitor. One placebo-controlled crossover study also showed that substance P increases sympathetic activity in the presence of combined ACE and DPP4 inhibition^18^. The unfavorable effects of this drug-drug interaction and the role of substance P are now subjects of an ongoing clinical trial in patients with T2DM (Effect of Chronic ACE and DPP4 Inhibition on Blood Pressure; NCT02130687). However, in SAVOR trial, there were no differences in heart or blood pressure changes after randomization according to baseline ACE inhibitor use in patients treated with saxagliptin or placebo (all *P* for interaction > 0.10)^19^. Nor were there any clinical consequences of baseline ACE inhibitor use on HHF alone (baseline ACE inhibitor: saxagliptin, 3.6% versus placebo, 3.1%; HR, 1.19; 95% CI, 0.95–1.49; *P* = 0.14 in comparison with no baseline ACE inhibitor: saxagliptin, 3.3% versus placebo, 2.4%; HR, 1.39; 95% CI, 1.06–1.83, *P* = 0.02; *P* for interaction = 0.38)^19^. Our study showed that the use of sitagliptin was associated with an increased risk of HHF in patients with T2DM receiving dialysis, especially in those without ACE inhibitors treatment. Longer duration and prospective studies are needed to prove these findings and effects. Third, because post-hoc analyses of data obtained from the SAVOR trial showed that the increased risk of HF was mainly found in patients with elevated NT-proBNP baseline levels or prior HF^6^, we examined the risk of HHF among patients with or without prior HF in this study. Similar to the SAVOR trial, our study found that the risk of HHF was higher among T2DM patients with previous HF. Moreover, glycemic control correlated not only with micro and macrovascular complications, but also with new-onset HF, supporting leading to the long-held assumption that reducing HbA1c with glucose-lowering drugs also reduces CV events and HF^20^,^21^. Theoretically, patients undergoing peritoneal dialysis experience higher glucose exposure from peritoneal dialysate compared with patients receiving hemodialysis, which may lead to a higher risk of HHF among these populations. However, we found that the risk of HHF after taking sitagliptin was higher among T2DM patients on hemodialysis rather than patients receiving peritoneal dialysis. This may be due to the small sample size in the peritoneal dialysis group.

Our study had several strengths. First, this was the first nationwide, population-based study to evaluate the risk of HHF of sitagliptin therapy in T2DM patients with ESRD on dialysis. Second, the use of the administrative database prevented under-reporting of medical visits^22^. Third, the nationwide population-based study design was highly representative of the general population and therefore prevented selection bias. Fourth, in our study, we adjusted for multiple confounding factors including the Taiwan comorbidity index, which is a better index for mortality prediction in Taiwanese patients receiving dialysis when compared with US renal data system index^23^.

However, several limitations of our study should be acknowledged. First, the lack of independent adjudication of HF commonly used in clinical trials might reduce the reliability of our findings. Although the NHI program regularly conducts expert reviews of patient charts to randomly confirm claims from all hospitals, bias may arise from miscoding and misclassification. However, the diagnosis in the NHIRD has been previously validated^24^,^25^,^26^. Second, several potential confounding factors for decompensated HF (e.g., body weight and caloric and salt intake), smoking status, and laboratory results were not available in the claim database. Third, this study included only Taiwanese patients who had different comorbidity patterns when compared to Caucasian patients with ESRD; therefore, the results might not be generalizable to other populations. Finally, as all patients with ESRD enrolled in our study were exposed to sitagliptin, the risk of HHF after exposure to other DPP-4 inhibitors requires further assessment.

In conclusion, sitagliptin use was associated with an increased risk of HHF in patients with T2DM receiving dialysis, especially in those without severe hypoglycemia, without ACE inhibitors treatment, with prior heart failure or receiving hemodialysis. In addition, there was a significant trend towards a higher associated risk of HHF as the dose of sitagliptin increased. Despite the enrollment and retention challenges inherent in studying therapies in dialysis patients, further assessment of the safety after using DPP4 inhibitors in patients with T2DM and ESRD on dialysis is required.

## Methods

The Taiwan National Health Insurance (NHI) program has offered comprehensive, universal health insurance to all residents of Taiwan since 1996 and covers more than 99% of the Taiwanese population (http://nhird.nhri.org.tw/en/Background.html). The National Health Research Institutes (NHRI) was commissioned to construct and maintain the National Health Insurance Research Database (NHIRD), which involved annual reimbursement claim data that was obtained from the Taiwan NHI program. All personal identification information was encoded to protect patient privacy before being released for research. The NHRI has created an anonymous identification number system that links each claimant’s demographic information to the NHIRD. The NHIRD uses the International Classification of Diseases, Ninth Revision, Clinical Modification (ICD-9-CM) to define disease diagnoses based on outpatient and inpatient data. The Registry of Catastrophic Illnesses Patient Database (RCIPD) is a subset of the NHIRD and eligible patients can apply for catastrophic illness certificates. If the claims are approved, patients are exempted from copayment of related medical costs. Both outpatient and inpatient claims of beneficiaries with a catastrophic illness certificate are collected in the RCIPD (http://www.nhi.gov.tw/webdata/webdata.aspx?menu¼&menu_id¼&wd_id¼&webdata_id¼ 3180). In this study, the history of ESRD was collected from the RCIPD. This study was approved by the Ethics Review Board at China Medical University (CMU-REC-101–012).

### Study population

We selected individuals with type 2 diabetes (ICD-9-CM 250) and newly diagnosed ESRD (ICD-9-CM 585) at the baseline from the RCIPD between 2000 and 2011. This was used to establish a population-based retrospective cohort study. Individuals with type I diabetes (ICD-9-CM 250.01, 250.03, 250.11, 250.13, 250.21, 250.23, 250.31, 250.33, 250.41, 250.43, 250.51, 250.53, 250.61, 250.63, 250.71, 250.73, 250.81, 250.83, 250.91, 250.93) and patients who had taken metformin, acarbose, saxagliptin, vildagliptin, linagliptin, or thiazolidinediones were excluded. The index date of the sitagliptin cohort was set as the first date of taking sitagliptin, and the follow-up was terminated when the patient developed HHF (which was ascertained by the ICD-9-CM 398.91, 425, 428, 402.x1, 404.x1, and 404.x3 in the first position of the hospital discharge diagnoses), when the patient withdrew from the insurance system, or on 31^st^ December 2011. We randomly selected a control cohort to match each case from the eligible source population by using propensity score matching method; these were individually matched for sex, age, duration of T2DM, hypertension medications (ARB, ACEI, calcium channel blockers, α-blockers, β-blockers, diuretics), use of statin and aspirin, use of other diabetes medications (sulfonylureas, glinides, and insulin), atherosclerotic heart disease, congestive heart failure and chronic obstructive pulmonary disease at a ratio of 1:4.

We systematically identified any risk factors for HHF as potential confounding factors, as defined by the following diagnoses recorded between January 1, 2000, and the index date: hypertension (ICD-9-CM 401–405), hyperlipidemia (ICD-9 CM 272), severe hypoglycemia, defined as two or more episodes of hypoglycemia (ICD-9-CM 251.0–251.2,775.6) requiring admission, and the Taiwan comorbidity index. Because Chinese patients with ESRD have different comorbidity patterns than Caucasian patients, we used the Taiwan comorbidity index, which demonstrates a better reclassification for mortality prediction in Taiwanese dialysis patients compared with that seen in the US renal data system index^23^. The Taiwan comorbidity index includes 10 comorbid conditions: diabetes (ICD-9-CM 250.xx, 357.2, 362.0x, and 366.41), atherosclerotic heart disease (ASHD, ICD-9-CM 410–414, V45.81, and V45.82), congestive heart failure (CHF, ICD-9-CM 398.91, 425, 428, 402.x1, 404.x1, and 404.x3), cerebrovascular accident or transient ischemic attack (CVA/TIA, ICD-9-CM 430–438), peripheral vascular disease (PVD, ICD-9-CM 440–444, 447, 451–453, and 557), chronic obstructive pulmonary disease (COPD, ICD-9-CM 491–494; 496; 510), gastrointestinal bleeding (GI bleeding, ICD-9-CM 456.0–456.2; 530.7; 531–534; 569.84–569.85; 578), liver disease (ICD-9-CM 570–571; 572.1, 572.4; 573.1–573.3; V42.7), dysrhythmia (ICD-9-CM: 426–427; V45.0; V53.3), and cancer (ICD-9-CM 140–172; 174–208; 230–231; 233–234). We calculated the mean comorbidity index score of both cohorts using the following comorbidity-related weight assignments: a weight of 1 assigned to ASHD; 2 to PVD and GI bleeding; 3 to diabetes, CHF, COPD and dysrhythmia; 4 to CVA/TIA and liver disease; and 6 to cancer. The diagnosis in the NHIRD has been previously validated^24^,^25^,^26^. We also identified several medication treatments as potential confounding factors, as defined by the following drugs recorded during the following period: angiotensin-converting-enzyme inhibitor (ACEI), angiotensin receptor blockers (ARB), calcium channel blockers (CCB), α-blockers, β-blockers, diuretics, statins, aspirin, sulfonylureas, glinides, and insulin.

### Statistical analysis

The mean and standard deviation for continuous variables and the number and percentage for category variables were used to describe the distribution of the cohorts. To test the difference between the cohorts, Student’s t test and the chi-square test were used for continuous and category variables, respectively. The total incidence and demography specific incidence of developing HHF was calculated per 10000 person-years. The Cox proportional hazards regression models, using both a crude model and a model adjusted for potential confounding factors, were used to estimate the hazard ratios (HRs) and confidence intervals (CIs) for the cohorts. Sensitivity analysis identifying sub-populations with a greater susceptibility was also performed by using the Cox proportional hazards regression model.

We used the defined daily dose (DDD) per year to quantify the average dose of sitagliptin (the Anatomical Therapeutic Chemical codes: A10BH01, A10BH02, A10BH03, and A10BH05). DDD is a technical unit used to measure drug consumption (WHO Collaborating Centre, 2003). The definition of DDD is the assumed average maintenance dose per day for a drug that is used for its main indication in adults. The defined daily dose is a unit of measurement and does not necessarily reflect the recommended or prescribed daily dose. Based on DDD, we established four categories of dose exposure. These were non exposure, low dose exposure (<180 DDD per year), intermediate dose exposure (180–359 DDD per year) and high dose exposure (≥360 DDD per year), which were then used to evaluate the effect of exposure dose on the occurrence of HHF. Data management and analysis were carried out with SAS 9.1 software (SAS Institute, Cary, NC, USA) and the incidence curve was drawn by using R software (R Foundation for Statistical Computing, Vienna, Austria). The significance level was set at a *p*-value of less than 0.05 for two-sided testing.

## Additional Information

**How to cite this article**: Hung, Y.-C. *et al*. Sitagliptin and risk of heart failure hospitalization in patients with type 2 diabetes on dialysis: A population-based cohort study. *Sci. Rep.* **6**, 30499; doi: 10.1038/srep30499 (2016).

## Figures and Tables

**Table 1:** Demographic data of the study cohorts at baseline.

Variable	ESRD		*p*-value
	Control cohort N = 3480 (%)	Sitagliptin cohort N = 870 (%)	
Age, years (SD)	65.5 (11.4)	65.3 (11.2)	0.75
<45	124 (3.6)	30 (3.4)	
45-64	1535 (44.1)	395 (45.4)	
≥65	1821 (52.3)	445 (51.1)	
Sex			0.82
Female	1691 (48.6)	419 (48.2)	
Male	1789 (51.4)	451 (51.8)	
Type of dialysis			<0.0001
HD only	3114 (89.5)	728 (83.7)	
PD only	62 (1.8)	41 (4.7)	
Both used	304 (8.7)	101 (11.6)	
DM duration, years (SD)	9.2 (3.3)	9.2 (3.3)	0.85
Follow-up duration, years (SD)	1.2 (0.8)	1.0 (0.8)	<0.0001
Taiwan comorbidity index			
Mean (SD)	10.6 (5.7)	10.4 (5.8)	0.20
ASHD	2340 (67.2)	579 (66.6)	0.70
CHF	1906 (54.8)	472 (54.3)	0.78
CVA/TIA	1622 (46.6)	384 (44.1)	0.19
PVD	1368 (39.3)	343 (39.4)	0.95
COPD	1272 (36.6)	321 (36.9)	0.85
GI bleeding	2286 (65.7)	554 (63.7)	0.27
Liver disease	1316 (37.8)	317 (36.4)	0.45
Dysrhythmia	886 (25.5)	226 (26.0)	0.75
Cancer	572 (16.4)	130 (14.9)	0.28
Hypertension	3469 (99.7)	866 (99.5)	0.52
Hypertension Medication			
ACEI	677 (19.5)	172 (19.8)	0.83
ARB	1474 (42.4)	367 (42.2)	0.93
α-blocker	503 (14.5)	121 (13.9)	0.68
β-blocker	1039 (29.9)	253 (29.1)	0.65
CCB	2463 (70.8)	603 (69.3)	0.40
Diuretics	1162 (33.4)	285 (32.8)	0.72
Hyperlipidemia	2667 (76.6)	671 (77.1)	0.76
Statin	1019 (29.3)	266 (30.6)	0.45
Aspirin	1637 (47.0)	386 (44.4)	0.16
Severe hypoglycemia	490 (14.1)	117 (13.4)	0.63
Other antidiabetic agents			
SU	964 (27.7)	248 (28.5)	0.64
Glinide	1305 (37.5)	341 (39.2)	0.36
Insulin	2040 (58.6)	487 (56.0)	0.16

ASHD, atherosclerotic heart disease; CHF, congestive heart failure; CVA/TIA, cerebrovascular accident or transient ischemic attack; PVD, peripheral vascular disease; COPD, chronic obstructive pulmonary disease; GI bleeding, gastrointestinal bleeding;

ACEI, angiotensin-converting-enzyme inhibitor; ARB, angiotensin receptor blockers; CCB, calcium channel blockers; SU, sulfonylureas;

HD: hemodialysis; PD: peritoneal dialysis.

**Table 2:** Incidence of heart failure hospitalization according to exposure of daily dose from the study cohorts.

Variable	N	Event	PYs	Rate	Crude HR (95% CI)	Adjusted HR (95% CI)
Sitagliptin users						
No	3480	309	4098	754	ref	ref
Yes	870	103	911	1130	1.49(1.2–1.87)	1.52(1.21–1.90)
DDD						
None	3480	309	4098	754	ref	ref
Low	674	74	740	1000	1.32(1.02–1.7)	1.35(1.04–1.74)
Intermediate	149	22	133	1653	2.18(1.42–3.37)	2.16(1.40–3.35)
High	47	7	38	1848	2.44(1.15–5.17)	2.57(1.21–5.47)
*p* for trend					<0.0001	<0.0001

The model was adjusted for age, sex, type of dialysis, DM duration, Taiwan comorbidity index, ACEI, ARB, α-blocker, CCB, diuretics, statins, aspirin, severe hypoglycemia, SU, glinide, and insulin.

DDD, defined daily dose; PYs, person-years; CI, confidence interval; HR, hazard ratio.

Low dose exposure, <180 DDD per year; intermediate dose exposure, 180–359 DDD per year; high dose exposure, ≥360 DDD per year.

**Table 3:** Risk of heart failure hospitalization with sitagliptin or placebo in patients with or without baseline risk factors (severe hypoglycemia, use of ACEI, prior heart failure, or type of dialysis).

Variable	Control cohort			Sitagliptin cohort			Adjusted HR (95% CI)
	Event	PYs	Rate	Event	PYs	Rate	
Severe hypoglycemia							
No	257	3539	726.2	87	803	1083	1.51(1.18–1.93)
Yes	52	559	930	16	108	1483	1.50(0.84–2.69)
Use of ACEI							
No	225	3098	726	80	683	1172	1.61(1.24–2.08)
Yes	84	1000	840	23	229	1006	1.26(0.78–2.03)
Prior HF							
No	71	1918	370	23	448	513	1.37(0.85–2.21)
Yes	238	2180	1092	80	463	1729	1.54(1.19–1.98)
Type of dialysis							
HD only	271	3676	737	87	744	1169	1.54(1.21–1.96)
PD only	3	69	438	2	46	439	—
Both used	35	353	991	14	122	1152	1.64(0.86–3.14)

The model was adjusted for age, sex, type of dialysis, DM duration, prior HF, ACEI, ARB, α-blocker, CCB, diuretics, statins, aspirin, severe hypoglycemia, SU, glinides, and insulin.

HD: hemodialysis; PD: peritoneal dialysis.
